# Assessing the impact of physical activity on reducing depressive symptoms: a rapid review

**DOI:** 10.1186/s13102-024-00895-5

**Published:** 2024-05-08

**Authors:** Nadia Samsudin, Richard Peter Bailey, Francis Ries, Siti Nur Aafifah Binti Hashim, Janet Ann Fernandez

**Affiliations:** 1 Faculty of Social Sciences and Liberal Arts, UCSI University, Kuala Lumpur, Malaysia; 2 Department of Physical Education and Sport, Faculty of Educational Sciences, University of Seville, Seville, Spain

**Keywords:** Well-being, Mental health, Public health, Mental wellness, Health education

## Abstract

**Context:**

The prevalence of depression among teenagers is a significant issue worldwide, which calls for a thorough investigation of non-drug treatments. This expedited evaluation examines 24 specifically chosen studies to clarify the correlation between physical activity depression symptoms in teenagers, undertaken following PRISMA principles.

**Methods:**

A wide range of research methods, including longitudinal studies, surveys, and cross-sectional analyses, were used in different nations to understand the intricate relationship between physical activity, sedentary behaviours, and depression symptoms. The data-gathering methods included standardised questionnaires, accelerometer measurements, and self-report instruments.

**Findings:**

The review highlights the crucial significance of engaging in physical activity to alleviate depression symptoms. Improved self-esteem consistently acts as a crucial intermediary between participation in physical activity and decreased rates of depression. Engaging in physical activity is a safeguard, particularly for individuals with restricted access to physical activity. In contrast, a sedentary lifestyle greatly increases the probability of developing moderate to severe symptoms of depression. Gender differences are apparent, with females being disproportionately impacted by depression. There are strong connections between engaging in physical activity and reducing symptoms of depression, which can be observed in various situations, such as participating in team sports or engaging in leisure activities.

**Conclusion:**

This study provides insight into the potential of physical activity as a non-pharmacological approach to address adolescent depression. This highlights the significant impact of physical activity, which has important implications for public health programs aimed at improving the mental well-being of adolescents by promoting physical activity. It is crucial to do additional research that considers gender-specific variations and various physical activity circumstances to enhance our comprehension of this important matter.

## Introduction

According to the World Health Organisation (WHO), adolescents are individuals between 10 and 19 years of age [1]. Profound changes in the body, hormones, brain, and intellectual abilities characterise adolescence. Adolescence is a period characterised by diverse phases of biological, psychological, and social growth that take place between puberty and attaining adult autonomy. During this phase, known as a critical period, adolescents are more susceptible to negative social effects and health repercussions [2, 3]. Adolescents commonly face various challenges, including decisions about education, physical appearance, job paths, aspirations, interactions with peers and love partners, and their political and social beliefs. Erikson [4] characterised adolescence as a phase marked by ambiguity and exploration regarding one’s identity and life trajectory.

Worldwide, it is estimated that roughly 14% of adolescents are affected by mental health difficulties, which contributes to over 13% of the overall disease burden in this age group [5]. Psychiatric problems are one of the primary contributors to the overall burden of health issues among young individuals, resulting in significant personal and societal expenses, both in the now and in the future [6]. Adolescents commonly experience depression, which has been worsened by the COVID-19 pandemic. Depression greatly diminishes their overall well-being and is marked by symptoms such as profound sadness, lack of interest, and disturbances in sleep and food [7].

Depression is the second most important contributor to the overall burden of disease among young people in Europe [2], and over 50% of those affected continue to experience it throughout their adult lives [8]. Quantitative research conducted in Europe has reached an agreement that there has been a rise in depression and other mental health disorders, including general anxiety and indications of stress [9, 10]. The qualitative data indicate a rise in despair, anxiety, and feelings of loneliness [11]. Similarly, the United States has also documented a substantial increase in the prevalence of depression among teenagers. The incidence of depression among adolescents increased nearly twofold, rising from 8.1% in 2009 to 15.8% in 2019 [12]. These findings align with other research conducted on adolescents in the Middle East, Africa, and Asia, suggesting the presence of a potential crisis in adolescent mental health [13].

The scientific literature has progressively supported the use of lifestyle management strategies, such as physical activity (PA), to prevent and treat mental health issues in adolescence [14, 15]. Abundant research has demonstrated the efficacy of consistent PA in diminishing depression symptoms in teenagers [16]. Regular PA is an effective method for controlling or avoiding mild to moderate depression. During PA, the body secretes many hormones, such as serotonin, dopamine, and endorphins. Serotonin regulates mood, sleep, memory, cognitive abilities, appetite, and digestion. Regular PA can enhance an individual’s mood and sleep quality, addressing the prevalent challenges faced by those with depression. Likewise, dopamine is linked to feelings of enjoyment, contentment, and drive. Elevated dopamine levels during PA result in sensations of happiness, bliss, and enhanced motivation and focus. Endorphins, also referred to as the body’s inherent analgesic, enhance relaxation and pleasure, hence diminishing stress. Moreover, PA might serve as a diversion from unproductive thoughts and everyday worries. When engaged with others, PA functions as a social support system and reduces sensations of being alone. This is because PA facilitates socialisation and fosters social connectedness. Prior research has shown that PA contributes to developing interpersonal connections and enhances self-worth in teenagers [17, 18]. Several studies indicate that PA may yield comparable outcomes to psychotherapy and pharmacological therapies while being more cost-effective, having fewer adverse effects, and providing broader health advantages [19, 20]. Several non-binding legal papers have endorsed this request, such as UNESCO’s ‘International Charter of Physical Education, PA and Sport’ [21] and the WHO’s ‘Global Action Plan on PA’ [22]. Nevertheless, the application of PA as a therapeutic approach for addressing mental health concerns in teenagers has not been well accepted [23, 24]. This can be attributed to the scarcity of empirical investigations and evaluations, namely those concentrating on juveniles and adolescents rather than adults [25].

It is worthwhile attempting some definitional clarity, as this broad area is prone to ambiguity [26]. The account of PA of key terms provided by Casperson et al. [26] have become highly influential and encompass both exercise-based and non-exercise-based activities. The former pertains to activities that are typically organised and involve significant energy expenditure, such as running and swimming. The latter pertains to both acute and persistent forms of PA, such as engaging in domestic tasks. Studies examining the effects of PA often prioritise exercise-based intensity levels, perhaps driven by the WHO objective of 60 min of moderate-to-vigorous activity per day in 2020 [27]. Although we recognise the importance of higher amounts of PA for health, we have considered both kinds in this review. A second distinction is, perhaps, even more relevant to this investigation, namely between physical inactivity and sedentariness [28]. These terms are sometimes conflated [29], but the consensus (and our working assumption for this study) is that physical inactivity and sedentary behaviour are distinct concepts with different health implications. While both are associated with chronic diseases, they have unique effects on cardiometabolic health [30]. National guidance typically follow the World Health Organisation’s [22] description of physical inactivity as simply insufficient levels of PA (less than 60 min of moderate to vigorous intensity activity daily). Sedentary behaviour is characterized by low energy expenditure, and can be an independent risk factor for metabolic issues, even in physically active individuals [31]. The stereotypical sedentary activity is, perhaps, television viewing, as it is a waking activity with an energy expenditure of 1.5 metabolic equivalent of task (METs) or less while in a sitting or reclining posture, which can coexist with high PA levels [28].

It is highly valuable to conduct research that critically evaluates the existing literature on the associations between PA and mental health to provide a comprehensive overview and assessment of the current scientific knowledge. This article presents an analysis of recent empirical studies.

## Methods and materials

This study employed a rapid reviewing methodology, incorporating several procedures utilised in systematic reviewing but modified to achieve a quicker and more diverse response [32]. This methodology enables the investigation of potential associations and contextual elements that could optimise the effectiveness of PA programs. (The study was registered with the National Institute for Health and Care Research. The registration ID for PROSPERO is CRD42023478488.)

Rapid reviewing has become an increasingly popular form of evidence synthesis in which mechanisms of the systematic review process are omitted or simplified to generate information more rapidly and/or for a more variegated response. They have been shown to generate similar conclusions to systematic reviews and constitute a recognised and valuable research method [33, 34] benefitting the scientific community by providing an incorporated, synthesised overview of the currently available evidence. Essentially, the rapid reviewing process adheres to the principles of systematic reviewing but balanced with great economies of time and flexibility of interests [35].

We searched for scholarly publications published in peer-reviewed journals from January 2018 to March 2023. This timeline was chosen to include the most recent studies relevant to this research topic and allow for an in-depth literature review in the years following significant developments in this field. The search was performed in three databases: Web of Science, SPORTdiscus, and the Psychology and Behavioural Sciences Collection. Some of the search terms employed include “physical activity* OR sport*,” “adolescent* OR youth*,” and “depress*.” Wildcard characters, such as asterisks (*) and question marks (?), accommodate word form differences and spellings.

In a systematic review, defined inclusion and exclusion criteria are essential for selecting relevant, high-quality literature that directly answers the research question. This section describes the criteria used for choosing publications on adolescent mental health i.e. depressive symptoms and physical activity. We established these criteria to maintain the scientific precision, integrity, and relevance of our review findings.

### Inclusion criteria

- Relevant, high-quality articles that are capable of answering the research question.
- Prioritising peer-reviewed studies to protect scientific integrity and credibility.
- To ensure consistency, accessibility, and practicality for the review committee, studies must only be in English.
- Focus on study into the depressive symptom impacts of PA on adolescents to ensure relevance to the research goal.

### Exclusion criteria

- Non-peer-reviewed resources.
- Studies that do not expressly address sports, physical activity, or mental health.
- Publications prior to 2018.
- Publications on serious mental illnesses, policy analysis, reviews, theoretical, and formative research.
- Interventions in clinical settings.

The search for relevant material was done in April 2023, covering the period from 2018 to March 2023. This timeframe was chosen to ensure that the review included the most recent and relevant studies available through March 2023. Given the principles of the rapid review technique, which emphasise timely synthesis of data, the goal was to include studies published as close to the review date as feasible to guarantee that the findings were current. While any papers released after March 2023 may not have been included in the search, it is believed that the timeframe chosen allowed for timely insights into the topic at hand and was consistent with the principles of the rapid review approach. Two independent reviewers examined the initial search results, assessing titles and abstracts for relevance to our predefined criteria. The reviewers resolved disagreements through discussion and, if necessary, consultation with additional reviewers. After iterating over the review-writing process, each reviewer reached a consensus on the content. Full texts of relevant articles during the initial screening were retrieved and examined for eligibility. Data extraction was carried out using a standardised form, which included crucial information such as study design, demographic characteristics, interventions, outcomes, and findings. The same reviewers extracted data independently and cross-checked it to verify correctness and consistency. The evidence was synthesised using a narrative method, combining research based on thematic similarities and comparing results across study types and populations.

**Table 1:** Research quality criteria

	Item	Score
**Study design**	Longitudinal design	2
	Cross-sectional - Adequately matched	1
	Cross-sectional - Limited information provided	0
**Frequency**	Multiple time points	2
	One-point	0
**Objective measures**	Present	2
	Not present	0
**Previous validation and/or reliability noted**	Studies used items from a previously validated scale	2
	Some information on reliability/validity of scales or items included	1
	Limited or no information on reliability/validity of scales or items included	0

The quality of the studies was assessed independently by two team members using a scale with a maximum score of 8. The classification was based on scores ranging from 0 to 8. Individual studies were thus categorised as low (scores 0 to 2), moderate (scores 3 to 6), or high (scores 7 to 8). Inter-rater reliability was evaluated by comparing the scores given by the different reviewers for each facet of the research quality rating scale to verify consistency. When Cohen’s kappa values fell between 0.81 and 1.00, signifying complete agreement in the quality evaluation area, the agreement levels were considered excellent. Table 1 displays the parameters used to ensure the quality of a product or process.

## Results

**Fig. 1:**
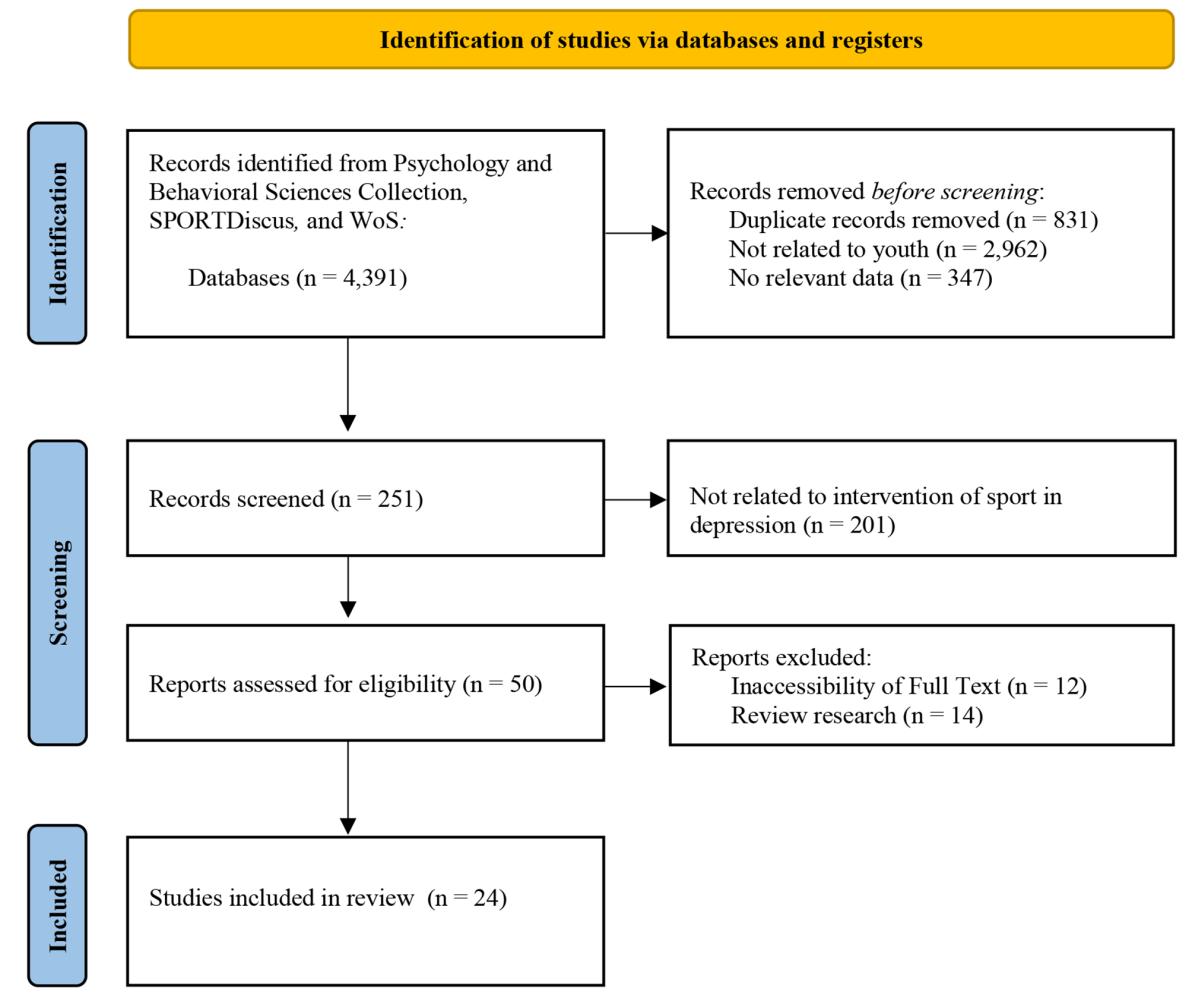
PRISMA output

This rapid review yielded 4,391 records. After screening and selection, 24 studies were included in the final review (see Fig. 1). The identified studies pointed to the intricate relationship between self-esteem, engagement in sports, volunteering activities, and depression. Higher levels of self-esteem were consistently associated with reduced rates of depression. Engaging in physical activities within the context of volunteering positively influenced self-esteem and acted as indirect pathways to lower depressive symptoms. This suggests that interventions promoting self-esteem through participation in socially orientated physical and activities may be beneficial in reducing depression among adolescents. While PA’s protective effect on depressive symptoms is evident, the optimal intensity and type of activity remain subjects of debate. Some studies highlighted the benefits of moderate PA, while others emphasised the importance of engaging in sport or PA with higher intensity. The diversity of findings underscores the need for tailored interventions that consider individual preferences and capacities.

The findings from these studies also reveal the interplay between PA, SB, and depressive symptoms. Excessive SB was not only linked to higher depressive symptomatology but also appeared to attenuate the benefits of PA. Adolescents who engaged in PA but counterbalanced it with extended periods of SB did not experience the same protective effect against depression. The selected studies underscore the pivotal role of PA and the detrimental impact of SB on depressive symptoms among adolescents. Regular engagement in PA and efforts to reduce SB can be essential components of mental health promotion strategies for this age group. Furthermore, fostering self-esteem through participation in sports and volunteering may provide a promising avenue to mitigate depressive symptoms. These findings collectively emphasise the importance of promoting an active and balanced lifestyle to support the mental well-being of adolescents worldwide. As determined by their quality rating scores, the incorporated studies were evaluated and classified. 14 studies (58.3%) were rated as moderate quality, whereas 10 studies (41.7%) were rated as high quality, out of a total of 24 studies that were examined. For reliable interpretation, evaluating the quality of the studies included is essential. Evidence is more reliable when it comes from high-quality studies, as opposed to moderate-quality ones. It is crucial that future research incorporate robust study designs, standardised assessments, and comprehensive reporting in order to increase methodological rigour. By incorporating these improvements, future research in the field can be more dependable and pertinent. Researchers have the ability to enhance decision-making and progress the understanding of a subject by ensuring the production of reliable evidence through the prioritisation of methodological integrity.

**Table 2:** Characteristics and quality assessment of eligible studies

Source / Country	Type of Study / Design	Objective / Sample	Methods / Measures	Instrument to measure Depression	Severity of Depression	Key Findings	Rating Score
Shen, Gu, Zhang, et al. (2022) / China	Survey / Cross-sectional design	To test a path model of TPB variables with PA and depressive symptoms. 792 students aged 15–18 years old	Participants completed questionnaires for TPB variables, PA for adolescents PAQ-A, and the short form of the Depression scale - CES-D).	Center for Epidemiological Studies-Depression scale	Majority of participants showed depressive symptoms.	- PA intention/behavior has a mediating effect on the TPB–depression relationship among adolescents. - TPB model can be used for designing and implementing PA interventions aimed to prevent depressive symptoms.	Moderate
Bohr, Boardman, & McQueen (2019) / USA	Longitudinal study / Cohort	To analyze adverse effect of actual/intended participation in contact sports during adolescence on cognition or depressive symptoms in early adulthood. 10,951 students aged 16 years old (wave I) and 29 years old (wave IV)	A subsample from the representative prospective cohort study Add Health was used to collect data through four waves (1994–2008). Modified CES-D scale was used to assess depression, suicide ideation, and suicide attempts at wave IV as a function of sport participation during wave I.	Center for Epidemiological Studies-Depression scale	Majority of participants showed depressive symptoms.	- Actual/intended participation in contact sports during adolescence did not adversely affect cognition or depressive symptoms in young adulthood.	High
Sun & Zhan (2021) / China	Online survey	To investigate the associations of PA, ST and SLP with depressive symptoms. 1,331 students aged 12–14 years old	Days of MVPA were assessed by HBSC questionnaire, together with ST and SLP. Depressive symptoms were measured using CDI.	Children’s Depression Inventory	Girls exhibited higher levels of depression symptoms compared to boy.	- Only ST was significantly and positively correlated with depressive symptoms. - Excessive ST may be detrimental to depression symptoms in adolescents.	Moderate
Xiao, Doig, Wu, et al. (2021) / China	Survey / Cross-sectional design	To analyze associations of sport participation with anxiety and depressive symptoms. 1,452 students aged 9–16 years old	Frequency of sport participation was self-reported, and PHQ-9 was used to measure the presence and severity of depression symptoms. Anxiety symptoms were measured using the seven-item GAD scale.	The nine-item Patient Health Questionnaire	Severe depression symptoms for those who never engage in sports activities.	- Engaging in sport participation was negatively associated with depression and anxiety.	High
Raudsepp & Vink (2019) / Estonia	Survey / Longitudinal study	To explore associations between PA, SD, and depressive symptoms. 16 students (girls) aged 12–15 years old (three waves over two years).	PA was assessed using the 3DPAR. Depressive symptoms were measured using CES-D. ISI scale assessed the severity of symptoms and consequences of insomnia.	Center for Epidemiological Studies-Depression scale	High depression levels were linked to less physical activity and more sleep disturbance.	- Initial levels of depressive symptoms predicted increase in SD and decrease in PA, and initial levels of SD predicted decrease in PA.	Moderate
Liu, Zhang, Hu et al. (2019) / China	Survey / Cohort	To identify combined patterns of MVPA and SSB, and to analyze the prevalence of different combined patterns and their correlations with depression, anxiety, and self-injurious behavior. 13,659 students (M_*age*_ = 15.18 ± 1.89 years) from NAEWIM-YRB	MVPA and SSB were assessed by the YRBS questionnaire, symptoms of depression by the 20-item CES-D scale, and anxiety symptoms using the 39-item MASC-2. Self-injurious behavior was measured using a 5-item subscale extracted from the HBICA inventory.	Center for Epidemiological Studies-Depression scale	Depression risk was higher in the group with low moderate to vigorous physical activity and high sedentary screen time.	- Significantly different probability of depression, anxiety and self-injurious behaviors were found, with boys being more at risk than girls. High MVPA/ low SSB subgroup showed significantly lower depression, anxiety, and self-injurious behaviors.	High
Cecchini, Fernandez-Rio, Mendez-Gimenez et al. (2020) / Spain	Survey	To examine the associations between PA levels, sedentary behaviours, and self-determined motivation and depressive symptoms. 714 students (girls) aged 15–18 years old.	Depressive symptoms were assessed using the six-item CES-D; PA with the Spanish version ‘seven-day recall’ of IPAQ, and situational (state) motivation towards an activity with SIMS scale.	Center for Epidemiological Studies-Depression scale	People with depression were often less active and more likely to be overweight.	- A strong association between MET-minutes/week and depressive symptoms, regardless of confounding factors such as age, smoking habits, and body mass index was found.	Moderate
Forte, McDowell, MacDonncha et al. (2020) / Ireland	Survey	To examine differences in depressive symptoms between adolescents reporting low, moderate, and high PA status and if depressive symptoms differed across PA status based on comorbidity status. 481 students (M_*age*_ = 15.1 ± 1.7 years)	The trait subscale of STAI assessed anxiety symptoms, and QIDS the depressive symptoms. PA was measured by the PACE+.	Quick Inventory of Depressive Symptomatology	Depressive symptoms were more severe in those with anxiety and low physical activity.	- Depressive symptoms were highest for low PA and lowest for high PA. - Among those with comorbid anxiety, compared to moderate PA, depressive symptoms were significantly higher for low and high PA. - Respondents with comorbid anxiety reported worse depressive symptoms.	Moderate
Slykerman, Thompson, Coomarasamy et al. (2019) / New Zealand	Prospective cohort study	To examine early adolescent PA and risk of later depressive symptoms. Mothers and their children: 467 students aged 16 years old	Depression at 16 was assessed using the CES-DC. Accelerometer measures of PA and sleep were measured at 11 years of age.	Center for Epidemiological Studies Depression Scale for Children	Minority had depression symptoms above the normal level, indicating moderate to severe depression.	- PA and sleep measured objectively and prospectively did not predict depressive symptoms at 16 years of age. - While PA and sleep may have health benefits, they did not reduce the risk of developing depression symptoms later in adolescence.	Moderate
Ogawa, Kitagawa, Fukushima et al. (2019) / Japan	Survey / Cross-sectional study	To examine the interactive effect of daily sleep duration and amount of PA on anxiety/depression in adolescents. 983 students aged 12–17 years old	Sleep duration was assessed in duration in hours and minutes. PA was estimated by using the level of participation in sports club activities at school. Anxiety and depressive symptoms in the previous 1 month were assessed by the GHQ-12 scale.	The 12-items General Health Questionnaire	Depression symptoms were higher in teenagers lacking both sleep and exercise.	- The main effects were statistically significant with positive impacts on anxiety/depression. - Anxiety/depression in adolescents with both adequate sleep and adequate PA may be at a similar level to that of adolescents with only one of these.	Moderate
Ma, Hagquist, & Løvheim (2020) / Sweden	Longitudinal survey	To explore how LTPA was associated with depressive symptoms among adolescents. 3,787 students aged 14–15 years old	Using binary logistic regression, the Swedish data collected as part of the Children of Immigrants: Longitudinal Survey in Four European Countries was analyzed.	The Children of Immigrants: Longitudinal Survey in Four European Countries	Depressive symptoms were less common among Swedish adolescents who regularly participated in physical activities during their leisure time	- Adolescents (both boys and girls) with LTPA on a daily, weekly, or monthly basis had substantially lower odds of often feeling depressed than those who were physically inactive. Girls had significantly higher odds of often feeling depressed.	High
Singh, Sharma, Raj et al. (2018) / India	Survey / Cross-sectional study	To examine a possible association between LTPA and depression 370 students aged under 25 years old	Mental health was assessed using CES-DC. LTPA was assessed by one item “How many hours a week do you usually exercise or play sports where you become breathless or have to sweat?”	Center for Epidemiological Studies-Depression scale	Higher levels of depressive symptoms are more common among students who engage in less than 4 h of leisure time physical activity.	- LTPA was found to be associated with lower rates of depression.	Moderate
Gu (2022) / China	Survey / Cohort	To examine the relationship between PA (including exercise activities and housework) and risk of depression.	CES-D was used to measure the individuals’ level of depression. Frequency, duration, and intensity of physical exercise were also assessed.	Center for Epidemiological Studies-Depression scale	Depression was significantly more likely among adolescents who reported intense physical exercise compared to those who reported little or no intense physical exercise.	- The frequency and duration of physical exercise participation are significantly and negatively associated with depression in adolescents. - Excessive exercise increases the likelihood of depression in adolescents.	High
Velazquez, Petresco, Pereira et al. (2022) / Brazil	Survey / Cohort	To explore associations between self-reported weekly PA and depressive symptoms. 7,405 students aged 14–16 years old.	Participants assessed were enrolled in the 8th through 11th grades in 101 public schools. PHQ-A and two items from PACE + were completed.	Patient Health Questionnaire-adolescents	Depressive symptoms were more common in those who exercised less frequently and vigorously compared to their peers.	- Participants with lower PACE + scores reported higher PHQ-A scores, indicating that teenagers who do not exercise frequently may have more depressive symptoms – with pronounced scores for both variables among girls.	High
Frömel, Jakubec, Groffik et al. (2020) / Czech Republic	Survey / Cohort	To study the associations between depressive symptoms, well-being, and different types of PA 596 students aged 15–19 years old	IPAQ-LF was used to identify the students’ PA; BFW in the modified and standardized Czechoslovak version was used to analyze the depression symptoms. The index of emotional well-being was also calculated. Weekly PA was objectively measured by pedometer.	Bern Questionnaire on Subjective Well-being	Higher depressive symptoms were associated with significantly less weekly recreational physical activity and lower well-being levels.	- The adolescents who were mostly involved in team sports showed fewer depression symptoms than those who were involved in other sports. - Neither boys nor girls with the most DS and the lowest well-being compensate for this emotional condition by engaging in PA in their after-school time or on the weekends.	Moderate
Xiang, Gu, Zhang et al. (2020) / China	Survey / Cross-sectional study	To examine the relations between different doses of PA, academic self-efficacy, and depression, and the direct and indirect relations of various doses of PA to depression through academic self-efficacy. 428 students (M_*age*_ = 13.7 ± 1.5 years)	PA was assessed using the LTPA scale. A 5-item academic self-efficacy scale was adapted to assess academic self-efficacy. The Chinese version of CES-D was used to assess the depressive symptoms.	Center for Epidemiological Studies-Depression scale	Majority of the respondents were suspected to have depression.	- The different doses of PA had significant indirect effects on depression through academic self-efficacy. Only MVPA but not LPA were significantly correlated with depression. - All forms/types of PA may contribute to adolescents’ mental health as well as their perceived academic self-efficacy.	High
Bélair, Kohen, Kingsbury et al. (2018) / Canada	Survey / Longitudinal study	To analyze the association between PA, sedentary activity and symptoms of depression and anxiety. 9,702 students aged 14–15 years old	The symptoms of depression and anxiety were measured with seven items taken from the Ontario Child Health Study. LTPA was measured regarding the frequency of participation. Sedentary activity was measured with the following question: ‘On average, how much time per day does he/ she watch T.V., videos or DVDs or play video games?’	Diagnostic and Statistical Manual of Mental Disorders	Depression was significantly associated with higher odds of being physically inactive and experiencing moderate symptoms.	- Participants with less than 1 day / week LTPA were at higher risk for being in higher depression and anxiety symptoms categories. - Sedentary activity was associated with increased odds of moderate and severe symptoms of depression and anxiety.	High
Bang, Won, & Park (2020) / USA	Longitudinal study / Cohort	To examine the relationships between school engagement, self-esteem, and depression and concurrently evaluate the effects of covariates, including sport participation, volunteering activity, and gender, on the study variables.	School engagement was measured, and self-esteem was measured by seven items, derived from the Rosenberg self-esteem scale. Depressive symptoms were measured by five items derived from the General Well-being Scale of the Current Health Insurance Study Mental Health Battery. Covariates were recoded as dummy variables. Volunteering activity was coded “1” if a respondent participated in any kind of volunteer activity	General Well-being Scale of the Current Health Insurance Study Mental Health Battery	Lower rates of depression were associated with higher levels of self-esteem.	- Higher levels of self-esteem were linked to lower rates of depression. - Higher levels of sport participation positively influenced self-esteem that was linked to low levels of depression. - Volunteering activity enhanced school engagement, which predicted self-esteem. - Volunteering activity was not directly associated with self-esteem that predicted lower depressive symptoms.	Moderate
Farren, Zhang, Gu et al. (2018) / USA	Survey	To investigate whether sedentary behavior and fitness-producing activity predicted depression in active adolescents over and above gender and fitness attributes. 249 students aged 12–13 years old	Accelerometers were used to assess SB and PA. Fitnessgram test items were used to assess HRF attributes	Center for Epidemiological Studies-Depression scale	For adolescents with depressive symptoms, staying physically active and maintaining good cardiorespiratory fitness can help counteract the negative effects of sedentary behavior.	- SB, fitness-producing activity, and attributes of HRF were all significant predictors of depression in active adolescents. - Fitness-producing activity significantly predicted depression in active adolescents beyond gender and achieved HRF.	Moderate
Holbrook, Voller, Castellini et al. (2022) / Italy	Survey	To explore whether gender, exercise frequency, and sport participation exerted a protective effect on the association between bullying and depressive symptoms. 4,829 students aged 13–21 years old	The data from EDIT project (Italy) was used. Exercise was assessed as the number of days in a typical week during which the respondent engaged in at least one hour of exercise. Depressive symptoms were questioned via six separate items (hopeless, depressed, useless, nervous, restless, and feeling as though everything was too hard).	Epidemiologia dell’Infortunistica Stradale survey	Females may be more affected by the depressive effects of bullying than males.	- A greater prevalence of bullying emerged among females with respect to males, with a greater tendency towards the phenomenon of social exclusion. - Significant interaction between bullying and gender was found in predicting depressive symptoms. - Exercise and depressive symptoms are significantly associated.	High
LaRocca, James, Rosenberg et al. (2022) / USA	Survey / Cohort	To examine the relationship between team sports participation, depression, and suicidal ideation. 46,537 students aged 14–18 years old	Students were enquired about team sports participation, depression, suicidal ideation, sexual orientation, and gender identity. Depression and suicidal ideation were assessed.	Healthy Kids Colorado Survey	LGBTQ youth who played team sports were less likely to experience depression.	- A statistically significant association between team sports participation and reduced likelihood for depression and suicidal ideation was found.	Moderate
Løvheim, Hartz, Thurston et al. (2018) / Norway	Survey / Cross-sectional study	To analyze the association between PA (sports club, gym, exercise independently and other organized PA) and symptoms of depression. 5,531 students aged 15–16 years old and 11,655 students aged 13–14 years old.	Symptoms of depression were measured, and the participants were asked how often they exercised or competed with a sports club, went to the gym, kept fit independently or practiced other kinds of organized PA. Parents’ level of education was measured separately for each parent. Smoking and alcohol use was also assessed.	Hopkins Symptom Checklist	Depression symptoms were less likely among individuals who engaged in physical activity through sports clubs, gyms, or independent exercise.	- The strength of the association between PA and symptoms of depression depends on the PA contexts. - Participation in PA in a sports club setting was related to fewer depressive symptoms.	Moderate
Chi & Wang (2022) / China	Survey / Cross-sectional study	To investigate the associations between sports participation and depression and anxiety. 1,714 students aged 12–17 years old	Depressive symptoms were assessed using PHQ-9. GAD-7 was used for assessing anxiety disorder. Sport participation was assessed using a single question. Sociodemographic factors were assessed using a self-reported questionnaire.	Nine-item Patient Health Questionnaire	Minority were reported normal severity of depressive symptoms.	- Students with less participation in sport-related activities had a greater likelihood of reporting depressive symptoms.	High
Kirklewski, Watson, & Lauckner (2023) / USA	Survey / Cohort	To examine the moderating effect of PA on the relationship between bullying and mental health among sexual and gender minority youth (LGBTTQ). 9,890 students aged 13–17 years old	Data from the LGBTQ National Teen Survey was analyzed. PA was measured using the Godin Leisure-Time Exercise Questionnaire. Depression was measured using the Kutcher Adolescent Depression Scale 11. Self-esteem was measured using 18 items from 3 scales (Rosenburg Self-Esteem Scale, Pearlin Mastery Scale, and Levenson Multidimensional Locus of Control Scale) Bullying was measured with 2 items.	Kutcher Adolescent Depression Scale 11	Depression is more prevalent in bullied youth who engage in less exercise.	- Depression was positively related to bullying. - Self-esteem was negatively related to bullying. - PA levels were negatively related to depression and positively related to self-esteem. - As PA increased, the relationships between bullying and depression and bullying and self-esteem became stronger.	High

Note: PA = Physical activity; SB = sedentary behavior; HRF = health related fitness; TPB = Theory of Planned Behavior; PAQ-A = Physical Activity Questionnaire for Adolescents; CED-D = Center for Epidemiological Studies-Depression scale; Add Health = National Longitudinal Study of Adolescent to Adult Health; ST = daily hours of screen-time; SLP = daily hours of sleep; MVPA = moderate-to-vigorous physical activity; HBSC = Health Behavior in School-aged Children; CDI = Children’s Depression Inventory; PHQ-9 = nine-item Patient Health Questionnaire; GAD-7 = Generalized Anxiety Disorder Scale; SD = sleep disturbance; 3DPAR = 3-Day Physical Activity Recall; ISI = Insomnia Severity Index; SSB = screen-based sedentary behavior; NAEWIM-YRB = National Assessment, Early-Warning and Intervention Model research on Youth Risk Behavior; YRBS = Youth Risk Behavior Survey; MASC-2 = Multidimensional Anxiety Scale for Children; HBICA = Health-Risk Behavior Inventory for Chinese Adolescents; IPAQ = International Physical Activity Questionnaire; MET = The Metabolic Equivalent of Task; SIMS = Situational Motivation Scale; BMI = body mass index; STAI = State-Trait Anxiety Inventory; QIDS = Quick Inventory of Depressive Symptomatology; PACE + = Patient-Centred Assessment and Counselling for Exercise Plus Nutrition; CAMHS = child and adolescent mental healthcare services; CES-DC = Center for Epidemiological Studies Depression Scale for Children; GHQ-12 = General Health Questionnaire; LTPA = Leisure Time Physical Activity; PHQ-A = Patient Health Questionnaire Adolescent; IPAQ-LF = The International Physical Activity Questionnaire—Long Form; BFW = Bern Questionnaire on Subjective Well-being; EDIT = Epidemiologia dell’Infortunistica Stradale; PHQ-9 = Patient Health Questionnaire

## Discussion

Major depressive illness has the highest prevalence of psychiatric issues among adolescents [36]. Due to its connection with adolescent suicide, it has been referred to as a “silent killer” that specifically affects susceptible young individuals [37]. In recent years, there has been a rise in the occurrence of depression in adolescence, prompting numerous international and national organisations to emphasise the immediate necessity for interventions aimed at preventing and treating this condition [38, 39]. The present review is situated in the context of a growing recognition of the potentially beneficial influence of PA on the mental well-being of adolescents. Nevertheless, the reasons and magnitude of these impacts remain relatively unclear and perhaps subject to controversy [40]. The disparity between assertions and the relatively antiquated state of many influential narratives implies the need for additional investigation.

The nature and magnitude of PA were identified as significant variables in numerous research included in this evaluation. Significantly, an increased risk was found at the lowest and highest activity intensity levels. One recent study analysed data from the China Family Panel Studies and found that individuals who reported exercising less frequently and for shorter durations had a considerably increased risk of depression [41]. Nevertheless, it was also documented that individuals who reported engaging in highly demanding levels of physical exertion were at a greater risk of experiencing depression. The results of Forte et al. [42] align with the findings of Gergelyfi et al. [43], which were not included in this review. The review and meta-analysis conducted by Carter et al. [44] on randomised controlled studies involving adults demonstrated that engaging in light and moderate PA had beneficial benefits for those with depression, while high levels of PA did not provide the same favourable outcomes. Nevertheless, studies have shown that engaging in any level of PA has contribute to the prevention and treatment of depression [17, 45]. This implies that engaging in even moderately low-intensity PA can be beneficial in reducing the likelihood of depression in adolescents. This is noteworthy because multiple research indicates that adolescents have a preference for physical activities with lower intensity [46].

Given that teenagers with depressive symptoms tend to engage in prolonged periods of inactivity [47, 48], targeting lower intensities as a feasible and meaningful objective for depressed young individuals is advisable. Additionally, it counteracts the detrimental impact of a sedentary lifestyle on depressed symptoms [49]. These findings might also support offering social and leisure activities instead of serious and competitive ones. Engaging in self-determined leisure activities gives individuals more autonomy in choosing how they participate, how long they participate, and how intense their participation is. This statement elucidates the discovery from one of the research in the review, which suggests an association between elevated levels of self-determined motivation and reduced levels of depressive symptoms [50]. Adolescents experience a boost in their sense of autonomy and intrinsic motivation when they have the freedom to select the type of PA in which they engage rather than feeling obligated or coerced to participate.

Articulating the exact reasons behind the unfavourable mental health outcomes shown in research linking increased levels of PA is a difficult task. Nevertheless, it is crucial to remember that the literature widely accepts the dangers of excessive PA, which may lead to the emergence of eating disorders, such as anorexia nervosa, bulimia nervosa, and binge eating disorder [42]. Given the heightened vulnerability of adolescents to negative body image, obsessive exercise can manifest as a significant issue. Excessive exercise can result in serious health problems, including repetitive strain injuries, fractures caused by stress, and harmful patterns of weight loss. Moreover, the perceived pressure arising from excessive competitiveness and time commitments can have adverse consequences [50]. It is important to emphasise that some types of competitive sports might result in negative mental health consequences as a result of bullying and other detrimental social behaviours [51]. Hence, it is imperative to use prudence when making oversimplified assertions that uniformly affirm sports’ beneficial impact on young individuals’ mental well-being [23].

Research has documented the advantages of sports clubs’ potentially social environments [52–54]. In some cases, participation in sports buffers against the effects of bullying and may prove a helpful strategy for increasing PA, positive peer interactions, and mood in adolescents [55]. Indeed, it is important to note that not all situations are identical. Kleppang et al. [56] contribute to this area of study by introducing the notion of ‘PA modalities’. They categorised involvement into 16 distinct groups, including ‘sports club’, ‘gym’, ‘independent’, and various combinations of these. Assuming that the role of the setting of PA is acknowledged as an important factor influencing mental health outcomes. Therefore, it is possible that distinguishing between different modes of engagement will be beneficial for future research. In their study, Kleppang et al. [56] found that teenagers who engaged in physical activities independently, such as swimming, jogging, or cycling alone, had a greater likelihood of experiencing depressive symptoms compared to those who participated in organised sports clubs. Due to its cross-sectional nature, it is impossible to ascertain the link’s direction. Nevertheless, additional investigation is required to classify the PA environment.

The research indicates that regular participation in exercise-based PA is most advantageous for promoting mental well-being [45, 57]. Chi and Wang [58] utilised ordinal logistic regression to investigate the association between sports involvement and depressive symptoms. Their findings indicated that persons with the lowest level of participation had the greatest probability of reporting depressed symptoms. However, the relationship between PA and mental well-being is complex. It can be affected by various circumstances, including the type of activity, its level of intensity, and the social environment in which it takes place [59]. However, it is logical to deduce that encouraging consistent PA among teenagers could significantly prevent and cure symptoms of depression [60].

There are multiple rationales for why PA promotes mental well-being overall and specifically alleviates symptoms of depression. The hypotheses might be categorised as ‘psychosocial’, ‘biological’, and ‘behavioural’ [61]. The psychosocial hypothesis emphasises the interpersonal advantages of various types of PA. Adolescents who frequently participate in sports and other prosocial activities are more inclined to establish favourable and enduring relationships with their peers [43, 59, 62]. Lubans et al. [61] suggested several factors, including perceived physical self-concept, competence, and physical appearance, could potentially influence the association. Two studies conducted with Chinese adolescents have examined this idea [39, 55]. The study conducted by Xiang et al. [45] provides evidence that various levels of PA significantly indirectly impact melancholy by improving academic self-efficacy. The researchers viewed this outcome as supporting the significance of PA for students’ academic performance, which would positively affect their mental well-being. Similarly, the study conducted by Shen et al. [60], which was based on the Theory of Planned Behaviour, provided evidence for the influence of psychosocial factors such as self-efficacy and positive attitudes. Furthermore, these data bolster assertions regarding the advantages of engaging in team-oriented pursuits, such as sports. A study conducted in the Czech Republic revealed that teenagers who engaged in team sports reported a lower number of depressive symptoms compared to their peers who participated in other types of PA [48]. This finding supports previous research conducted in different areas [62, 63].

It has been previously observed that engaging in sports can provide stronger protection against the negative impacts of bullying compared to general PA [55]. Undoubtedly, portraying athletics as a cure-all would be imprudent, and any impacts should be comprehended in the wider framework of teenage growth. One can glean an important insight from examining the phenomenon of bullying among sexual and gender minority kids [59]. The researchers discovered an inverse correlation between PA and depressed symptoms, as well as a direct correlation with self-esteem. However, subsequent data analysis indicated that these benefits may have been restricted to persons who did not experience bullying. Within this study, it was found that PA has a minimal impact on individuals who have experienced bullying. This underscores the crucial significance of creating an environment where minority or marginalised groups are embraced, and bullying is unequivocally condemned. When implementing such measures, it is reasonable to assume that potentially susceptible young individuals can experience comparable mental health advantages linked to sports engagement, just like any other person [54].

The review findings also supported the behavioural hypothesis, which is another account. The assertion posits that modifications to specific behaviours serve as intermediaries in the connections between mental health and PA. Raudsepp and Vink [65] conducted a study to investigate the long-term connections between PA, depressive symptoms, and sleep. They discovered that these three variables were interconnected. Specifically, they found that higher levels of depressive symptoms at the beginning of the study predicted sleep problems and reduced PA. Similarly, initial levels of sleep disorders predicted a decrease in PA. The reasons for these relationships remain uncertain.

One further potential reason for the negative relationship between PA and depression symptoms in teenagers is that engaging in PA may provide protection through neurological mechanisms [59, 61]. This topic was not addressed in the publications included in this review. As previously stated, this association could be attributed to the various hormones secreted by the body during PA. The hormones serotonin, endorphins, and dopamine act as natural antidepressants, improving mood, alertness, sleep, and motivation.

## Conclusion

These selected studies, conducted in various countries, provided valuable insights into the relationship between PA, sedentary behaviour (SB), and depressive symptoms in adolescents, offering a robust foundation for understanding this critical aspect of adolescent mental health. Regular PA was consistently found to protect against the development of depressive symptoms. Adolescents who participated in sports or engaged in PA demonstrated a reduced likelihood of experiencing depression. This positive correlation was noted in various countries, emphasising the global importance of PA in enhancing mental well-being. Notably, the frequency and duration of engaging in PA also had a significant impact. Adolescents who included PA in their daily or weekly routines had significantly reduced chances of experiencing frequent depressive symptoms. This discovery emphasises the significance of regular, sustainable PA as a preventive measure against depression during this crucial period of growth and development. In contrast, an excessive amount of SB has consistently been associated with a higher risk of experiencing moderate and severe depressive symptoms. The prevailing sedentary lifestyle among adolescents today has prompted concerns regarding its detrimental impact on mental well-being. These studies emphasise the significance of decreasing SB as a possible approach to alleviate depressive symptoms in adolescents.

Consistent with previous research, this review discovered a correlation between depressed symptoms and PA levels, indicating that symptoms were more severe in teenagers who were less physically active. Engaging in suitable PA enhances the emotional and physical well-being of adolescents. Significantly, medicine or other interventions cannot reproduce several attributes of PA. Multiple studies in this study corroborated the “psychosocial hypothesis,” which emphasises the psychological and social advantages of engaging in various types of PA, particularly sports. This highlights the need to guarantee access to inexpensive options for consistent and diverse physical activities. Moreover, these findings support the World Health Organisation’s objective of promoting 60 min of PA each day [64]. Nevertheless, it is crucial to recognise that engaging in moderate-to-vigorous physical activities may pose difficulties for certain teenagers who are struggling with depression. Therefore, directing attention towards reducing inactivity may be a more feasible objective. Unfortunately, multiple studies suggest that even these less ambitious goals are not being achieved [65, 66].

A limitation of this review, and of the field as a whole, is the relative ambiguity and inconsistency regarding measures of depression. The majority of research into lifestyle interventions, such as PA, has focused on mild-to-moderate depression [20], and that is consistent with general guidance [67]. However, as can be seen in Table 1, little detail was provided about the severity of depression in most studies, and this inevitably undermines judgements about the efficacy of interventions [67]. This seems an area warranting serious attention in subsequent research.

To summarise, the information reported in this review strengthens the persuasive argument for encouraging PA among adolescents from diverse backgrounds. An essential finding from multiple research in this review is that protection against depressive symptoms is not solely derived from PA itself but also from the social environment in which these activities take place. To enhance our young population’s mental health and overall well-being, we should create an atmosphere that promotes consistent and diverse PA. Striving for a better and happier future during adolescence is worth pursuing with enthusiasm and dedication.

## Data Availability

The datasets generated and analyzed during the current study are available upon reasonable request from the corresponding author.

## Abbreviations

WHO: World Health Organisation
PA: physical activity
SB: sedentary behaviour
